# MiRNAs as Players in Rhabdomyosarcoma Development

**DOI:** 10.3390/ijms20225818

**Published:** 2019-11-19

**Authors:** Patrizia Gasparini, Andrea Ferrari, Michela Casanova, Francesca Limido, Maura Massimino, Gabriella Sozzi, Orazio Fortunato

**Affiliations:** 1 Tumor Genomics Unit, Department of Research; Fondazione IRCCS Istituto Nazionale dei Tumori, Via Venezian 1, 20133 Milan, Italy; gabriella.sozzi@istitutotumori.mi.it; 2 Pediatric Oncology Unit; Fondazione IRCCS Istituto Nazionale dei Tumori, Via Venezian 1, 20133 Milan, Italy; andrea.ferrari@istitutotumori.mi.it (A.F.); michela.casanova@istitutotumori.mi.it (M.C.); francesca.limido@gmail.com (F.L.); maura.massimino@istitutotumori.mi.it (M.M.)

**Keywords:** rhabdomyosarcoma, microRNA, pediatric tumors

## Abstract

Rhabdomyosarcoma (RMS), the most common soft tissue sarcoma of childhood and adolescence, is a rare but aggressive malignancy that originates from immature mesenchymal cells committed to skeletal muscle differentiation. Although RMS is, generally, responsive to the modern multimodal therapeutic approaches, the prognosis of RMS depends on multiple variables and for some patients the outcome remains dismal. Further comprehension of the molecular and cellular biology of RMS would lead to identification of novel therapeutic targets. MicroRNAs (miRNAs) are small non-coding RNAs proved to function as key regulators of skeletal muscle cell fate determination and to play important roles in RMS pathogenesis. The purpose of this review is to better delineate the role of miRNAs as a biomarkers or functional leaders in RMS development, so to possibly elucidate some of RMS molecular mechanisms and potentially therapeutically target them to improve clinical management of pediatric RMS.

## 1. Overview RMS (Rhabdomyosarcoma)

Soft tissue sarcoma (STS) are rare malignant tumors that arise in connective tissues. STS accounts for approximately 7% of pediatric cancers and 1% of adult cancers [1]. Rhabdomyosarcoma (RMS), representing about 50% of all pediatric STS, is a typical embryonal tumor of childhood showing skeletal muscle differentiation (WHO STS 2013). Overall, RMS has a frequency of 4.3 million/year, two thirds of which are diagnosed in children <7 years. Although it occurs mainly in the pediatric age (0–10 years), RMS has a second peak of incidence in adolescence and young adulthood (15–30; AYA); in contrast, RMS is extremely rare in adults [2]. RMS is a heterogeneous tumor for histology, site of incidence, prognosis and, also, age. RMS has different histotypes: alveolar, embryonal, pleomorphic and spindle cell/sclerosing [3,4], but the most frequent in pediatric RMS are the embryonal (ERMS) and alveolar (ARMS). Both subtypes are characterized by distinct molecular profiles which play a decisive role in cancer pathogenesis and often delineates patients’ prognosis [3,5]. ERMS represents ~80% of all RMS cases and, generally, affects younger children (0–4 years) and arises in favorable sites such as neck, head and genito-urinary tract. Morphologically, tumor cells resemble embryonal skeletal muscle cells [3]. On the other hand, ARMS is a high-grade malignancy occurring mostly in adolescents and young adults, representing about ~20% of RMS cases [6,7]. Typically, it originates in the limbs and trunk, often with regional or metastatic lymph node involvement at time of diagnosis, and carries a significantly worse outcome [8]. Alveolar RMS is so called because tumor cells are arranged in a pattern forming small spaces or pseudo-alveoli.

Currently, multidisciplinary management, including chemotherapy and surgery with or without radiation, is considered the standard treatment of care for RMS. The 5-year survival rate of RMS has increased from 25% in 1970 up to 60% since 2000 [4,9]. While generally responsive to the modern multimodal therapeutic approaches including multidrug intensive chemotherapy, RMS remains a high grade malignant disease with a marked propensity to metastasize. The prognosis of RMS, however, depends on multiple variables, and for some patients the outcome remains dismal [7,10]. Moreover, patient’s age has emerged as a factor significantly influencing survival for RMS patients: 66.6% OS in children, while dropping to an average 38% OS in AYA.

In recent years, little improvements in oncologic outcome of patients with RMS has been observed and drug resistance and metastatic disease represent the two most common issues for therapy failure. Thus, there is an urgent need for more effective treatment regimens. To date, comprehensive molecular and genomic analysis of these tumors has produced substantial new insights into molecular cell biology, molecular cytogenetics and tumorigenesis of RMS, leading to a better understanding of RMS development at the molecular level. These advances may ultimately lead to: a) better clinical understanding; b) potentially develop safer and more potent targeted therapies; c) drive new initiatives to improve their final outcome.

The purpose of this review is to better delineate the role of miRNAs as a biomarker or functional leader in RMS development, so to possibly clarify some of RMS molecular mechanisms and potentially therapeutically target them to improve clinical management of pediatric RMS. Firstly, we will address the molecular cytogenetic characterization of RMS, as the genetic landscape plays a critical role in RMS tumorigenesis. Moreover, the involvement of miRNAs in normal myogenesis, identifying both critical muscle-specific miRNAs and those miRNAs not specific to muscle, will be described so to better comprehend the most critical miRNAs involved in this mechanism. Moreover, an overview the most relevant works of miRNAs identified as potential biomarker for RMS development will be presented, followed by a summary of studies that report miRNAs as functional players in RMS. Finally, for each section, limitations of the presented studies will be discussed so to possibly overcome problematic issues and and achieve better results.

### Molecular Cytogenetic Characterization of RMS

Genetic factors play an important role in the development and progression of RMS and although the majority of cases are sporadic and not associated with hereditary syndromes, there is yet a small proportion that are linked to congenital anomalies (Beckwith-Wiedemann syndrome), or are associated with particular familial syndromes (neurofibromatosis type I and Li-Fraumeni syndrome) [11,12,13]. Moreover, the malignant transformation of RMS also occurs cytogenetically due to the accumulation of somatic mutations by the acquisition of tumor-specific chromosomal aberrations [2]. All cytogenetic alterations observed in pediatric ERMS and ARMS are listed in Table 1.

**Figure 1:**
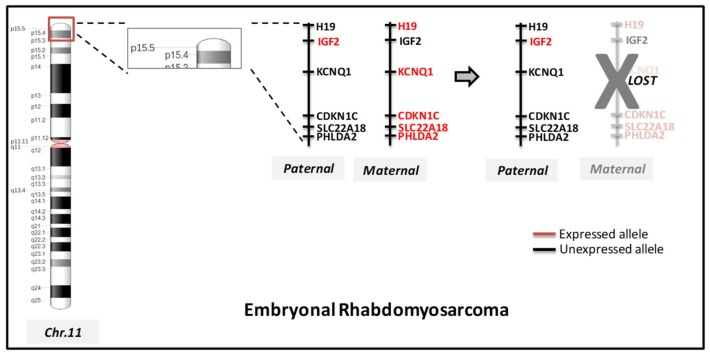
Allelic loss of imprinted region at 11p15.5. The chromosomal ideogram of 11p15.5 illustrates imprinting of genes alleles included in that specific region according to parent-of-origin. Particularly in embryonal RMS ERMS, the maternal allele is lost, through loss of heterozygosity, resulting in the presence of only the paternal allele.

As for chromosomal rearrangements, the embryonal subtype is mainly characterized by the presence of loss of heterozygosity (LOH) of the short arm of chromosome 11 (11p15.5) and 16q [14,15]. It has been demonstrated that the most common rearrangements in ERMS are the inactivation of the parental bias of chromosome 11p15 [16] (Figure 1). Novel complex chromosomal rearrangements in *PAX3* were rarely reported [17].

**Figure 2:**
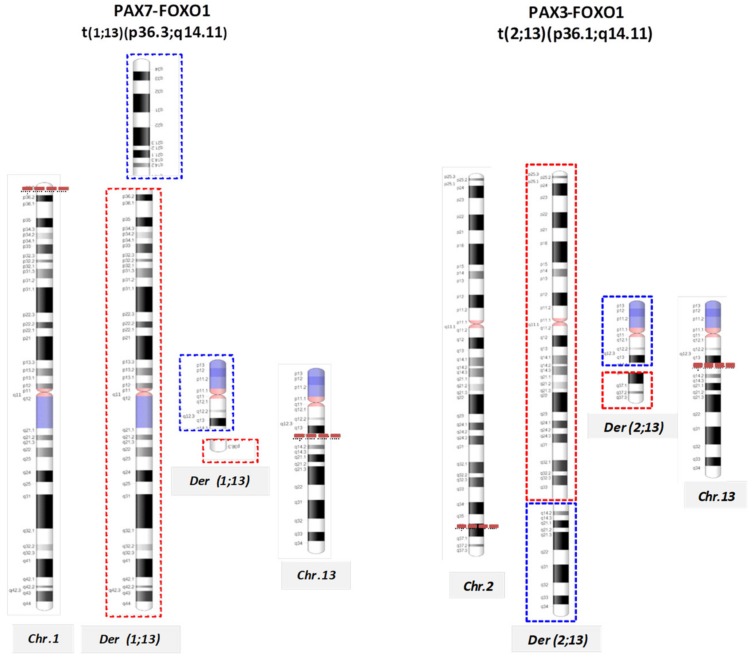
Chromosomal rearrangements in alveolar RMS ARMS. A diagram of t(1;13)(p36.3;q14.11) and t(2;13)(q35;q14) illustrates reciprocal translocations that generate fusion genes PAX7-FOXO1 and PAX3-FOXO1, respectively. The red and blue dotted lines delineate the derivative chromosomes: the red boxes represent regions of chromosome 1 (on the left) and chromosome 2 (on the right) involved in the rearrangement, while the blue ones illustrate the translocated region of chromosome 13 ( both on the left and right).

Approximately 80% of ARMS are cytogenetically characterized by chromosomal aberrations traslocating t(2;13)(q35;q14), respectively involving *PAX3* gene at 2q35 to the *FOXO1* gene at 13q14, generating a fusion transcript PAX3-FOXO1. Less frequently, the t(1;3)(q36;q14) translocation involves *PAX7*, on chromosome 1, and *FOXO1*, generating PAX7-FOXO1 fusion genes (Table 1; Figure 2) [18,19].

It has been demonstrated that the PAX3-FOXO1 fusion gene status significantly improves current risk stratification, as opposed to the presence of PAX7-FOXO1 [20]. Other rare variants involving the genes PAX3 and FOX1 have been reported and are listed in Table 1. Interestingly, about 20% of ARMS do not present FOXO1 rearrangements and are considered to be ‘fusion-negative’ (FN)-RMS, that have a clinical course similar to ERMS. Overall, ‘fusion-positive’ (FP)-RMS carry a low burden of somatic mutations but multiple genes were recurrently altered, including *NRAS*, *KRAS, HRAS, FGFR4, PIK3CA, CTNNB1, FBXW7* and *BCOR* [21]. Considering chromosome acquisitions or deletions, generally, ERMS are characterized by gains or losses of specific whole chromosomes, whereas ARMS display genomic amplification of specific chromosomal regions (Table 1) [2,22,23,24,25,26]. Several CGH studies have shown that ARMS tend to have fewer copy number variants than ERMS tumors [27,28], but that those alterations are responsible for tumor progression and proliferation [22].

The detection of any possible genetic alteration is critical for the diagnosis, prognosis and, at times, therapeutic and clinical management of RMS. Currently, conventional methods to detect chromosomal rearrangements in routine diagnostics, in particular in pediatric soft tissue sarcomas, rely on fluorescence in situ hybridization (FISH), immunohistochemistry (IHC) and/or quantitative real-time polymerase chain reaction (RT-PCR). All these methods are highly sensitive but with a low-throughput and only test for the presence of a single gene alteration (mutation, fusion gene or protein), resulting in a lengthy, tissue consuming and, most of all, costly diagnostics course investigations [29]. In contrast to the traditional techniques, large-scale sequencing, such as next generation sequencing (NGS) can provide high-resolution fusion gene and protein detection, while evaluating hundreds of genes in a single analysis with a faster turn-around time, lower costs and small amount of sample material [29].

Overall, although several tumor causative genes have been identified by molecular cytogenetics, a detailed understanding of the molecular mechanisms underlying RMS development has not yet been achieved. MicroRNAs (miRNAs) are small non-coding RNAs with a proved role as key regulators of skeletal muscle cell fate determination. Recent studies have suggested miRNAs as key players in RMS and to be dysregulated in ARMS and ERMS [3]. Importantly, cytogenetic rearrangements are associated with aberrant expression of miRNAs in cancer cells, as miRNAs are deregulated subsequent to chromosomal alterations [30]. Moreover, the presence of specific oncogenic fusion genes in several sarcomas, including RMS, is an appropriate model system critical to dissect the complex miR-modulated pathways that originate these peculiar cancers [31]. Detecting such abnormalities is critical for diagnosis, prognosis and therapeutical regimen of these malignancies, mostly due to the increasing knowledge of associations of deregulation at microRNA loci [30,31].

## 2. miRNAs (MicroRNAs) Involvement in Myogenesis

Muscle tissue originates from a process called myogenesis, in which numerous genes involved in the regulation of the different stages of cell maturation are involved. The regulation pathway that determines muscle development is due to a specific class of transcription factors, called myogenic regulation factors (MRFs) [32]. The expression of MRFs, which include myogenic factor 5 (*myf5*), *myoD, myogenin* and *MRF4*, is restricted to the muscle lineage resulting in the activation of a cascade of events that lead to the formation of mature muscle fibers. The complex process includes regulators upstream of the MRF such as sine oculis-related homebox (*SIX*), their eyes-absent cofactors (*EYA*) and Paired box transcription factors (*PAX3* and *PAX7*). MiRNAs play a pivotal role in modulating post-transcription of all these processes, proving to be critical in the development of normal skeletal muscle tissue [33].

In recent years, the typical pattern of temporal expression of tissue-specific miRNAs has been studied during the process of development of skeletal muscle tissue by microarray analysis or sequencing [34]. Differentially modulated miRNAs were identified in C2C12 myoblasts when induced to differentiate in horse serum [34]. In particular, miR-133a-1, miR-133a-2, miR-133-b and miR-206 were the most significantly upregulated miRNAs, confirming their fundamental role in myogenetic differentiation [35,36]. MiRNAs in muscle tissue are classified in: a) myo-miRNAs, muscle specific; b) non-myomiRNAs, present in skeletal muscle and in cardiac muscle.

### 2.1. Myo-miRNAs

A list of the most relevant myo-miRS and their target genes with their function is given in Table 2.

MiR-1/206 cluster is categorized as a muscle-specific miR and consists of six different miRNAs located on three different chromosomes [35,37]. Myogenic transcriptor factors such as *MyoD*, *MEF2* and *SRF* directly regulate the expression of miR-1 and miR-133a in skeletal muscle during myogenesis, while the expression of miR-206 is controlled by *MyoD* and *MyoG* [38,39]. The crucial position of miRNAs in the regulation process of myogenesis was also corroborated with in vivo models [40].

MiR-1 and miR-133 are crucial for regulation of the proliferation and differentiation of skeletal muscle cells and act at the histone deacetylase 4 level (*HDAC4*) and *SRF* respectively, thus establishing negative feedback for myocyte differentiation [35]. MiR-1 guides a decreased cardiomyocyte proliferation, attributable to the decreased expression of *HAND2* [38]. Moreover, the genetic interaction between miR-133a and serum response factor (*SRF*) determines the upregulation of miR-133a, resulting in repression of *SRF* and constituting a negative feedback cycle. However, the primary function of miR-133a is to promote proliferation and inhibit differentiation, while for miR-1 it is induction of differentiation of mesodermal progenitors towards muscle lineage. Paradoxically, miR-133a and miR-1 show opposite effects on skeletal muscle development, although they derive from the same polycistronic transcript of miRNA. Therefore, miR-1 and miR-133 have a specific role in proliferation and in the differentiation of muscle cells in an antagonistic way, with the equilibrium altered in one way or another by additional modulators of gene expression [41]. As for miR-206, it represses the expression of the p180 subunit of DNA polymerase α1, *Pax7* [42], follistatin-like 1 or utrophin [43], thus suppressing muscle cell proliferation through inhibition of DNA synthesis. MiR-206 plays a critical role of a strong inducer of muscle cell differentiation.

### 2.2. Non-myomiRNAs

In addition to muscle-specific miRNAs, numerous non-specific miRNAs exists and are involved in the regulation of myogenesis. Non-myomiRs regulate muscle proliferation and differentiation through the repression of target genes involved in multiple processes (Table 3) [44,45,46,47,48,49].

In details, when miR-27b is up-regulated, an improper migration and an early differentiation of myoblasts at the level of the PAX3 [44] protein is induced at the beginning of myogenesis. Similarly, miR-26a [45] and miR-214 [46,47] also act as promoters of myogenesis, directly acting on the inhibitor *EZH2*. The two miRs differ from each other in when they are expressed: miR-214 is up-regulated by *MyoD/MyoG* only once muscle differentiation has begun, whereas miR-26a gradually increases throughout myogenesis. Inhibition of the homebox protein A11 (*HOX11*) by miR-181 is another step in muscle differentiation [48]. Moreover, *HOX11*, target gene for miR-181, low expression leads to an increase in *MyoD* and a correct differentiation in muscle cells. As miR-181 is up-regulated during muscle development, it is down-regulated in adult skeletal muscle tissue [50]. In contrast, miR-669a and miR-669q are expressed in cardiac muscle to prevent differentiation in skeletal muscle from the outset, inhibiting *MyoD* and its molecular targets, thus ensuring that skeletal muscle myogenesis occurs at the correct points [49].

## 3. miRNAs as Biomarker for RMS Development

Most of muscle-specific processes can be modulated post-transcriptionally by miRNAs, as their deregulation has been implicated in the initial, progression and metastatization phases, suggesting their potential as biomarkers in RMS development. Moreover, myomiRs have been shown to be significantly deregulated in RMS [51], and since myomiRs are essential for the differentiation of skeletal muscle tissue, their downregulation could be one of the factors responsible for the dedifferentiation of their phenotype in RMS [52]. Rao et al. reported that miR-1 overexpression in RD cell line (ERMS) determines the typical gene expression of muscle tissue and cell cycle arrest, while miR-133a decreases the expression of muscle markers [41]. Similarly, miR-1 and miR-133a have the same distinct role in normal muscle differentiation. MiRNAs regulation can be modified according to their cellular context; therefore, an inhibitory effect on cell growth can be observed following a forced expression of miR-1 or miR-206 in RMS cell line, both in vitro and in vivo [53,54]. The induction of miR-1/206 precursor led to a decrease in myogenic differentiation and inhibition of tumorigenic potential. Furthermore, mRNA analysis, before and after transfection of miR-206 in RD cells, revealed more than 700 modulated genes, including *c-MET* [54]. The *c-Met* downregulation by miR-1/206 resulted in a significant inhibition of RMS development, suggesting that c-Met targeting is one of the underlying mechanisms responsible for the development of RMS.

Particularly for RMS, miR-1 and mir-206 have been reported downregulated in RMS, both the embryonal and alveolar subtype. Specifically, PAX3 protein expression is observed repressed in ERMS, but not in ARMS. The antitumor capacity, determined by the ectopic expression of the miR-1/206 cluster in RMS, was further verified by the observation that these miRNAs directly regulate the expression of *CCND2*, a cell cycle gene [55]. Overexpression of miR-1/206 showed a strong pro-myogenic effect in RMS cells and reduced *CCND2* transcription levels. Furthermore, miR-1/206 significantly reduces the expression of PAX3 protein in the ERMS cell line; however, it has not shown any effect on PAX3 protein levels in an ARMS cell line, suggesting that the PAX3-FOXO1 fusion gene present in ARMS might obstruct the regulation of PAX3 by those specific miRNAs [55]. All these findings highlight, once again, the importance of the cellular context in determining the response to miRNA modulation. Additionally, miR-22 a miRNA induced in physiological normal muscle differentiation, is down-regulated in RMS, and its replacement blocked tumor growth and dissemination in pre-clinical in vivo models [51].

Not only myomiRs, but also non-muscle specific miRNAs have been implicated in the regulation of RMS development. MiR-29 deregulation was reported in a small cohort of ARMS, associated with an inhibition of myogenesis [56], whereas miR-183 acts as an onco-miR in different types of cancer, including RMS, synovial sarcoma and colon cancer [57]. By analyzing a small group of five RMS tissues, mir-9 was observed to be over-expressed in ARMS compared to ERMS, resulting correlated to metastatic potentials [58]. Additionally, another small cohort of 15 RMS (seven ARMS and eight ERMS) identified 97 miRNAs differentially expressed in RMS compared to normal skeletal muscle [3].

Our recent work delineated the potential of miRNAs to discriminate miRNAs associated to age in RMS [59]. Age (> 10 years of age) is considered a critical unfavorable prognostic factor for RMS [8]. MiRNA analysis revealed 39 miRNAs increasing with age, as opposed to 20 miRNAs decreasing with age and enriched in pediatric RMS. Among all the detected miRNAs, miR-223 was associated with up-regulation of epithelial mesenchymal translation (EMT), possibly implicating its role in aggressiveness and inflammatory pathways of RMS in the adolescents and young adults. MiR-431 was correlated to myogenic differentiation and muscle metabolism, yet supporting its increase in pediatric RMS [59].

In conclusion, literature emphasize the importance of miRNAs as biomarker for the detection of RMS, but none of them address the discovery and identification of miRNAs differentially expressed at an early phase of RMS development. One important limitation of all studies describing any miRNA modulation in RMS is that the cellular origin of miRNAs in not taken in consideration. This way, the de-regulation of the identified miRNAs could possibly reflect changes in their cellular composition and not modulation in miRNAs expression within cancer cells. Moreover, being RMS a rare malignancy, not only the size of the sample series analyzed is small but also the specimen material, such as FFPE tissues, is very limited. Furthermore, in these studies, the absence of a larger RMS validation cohort, either internal or external to the institute, remains a major weakness as the results obtained cannot be confirmed. Creating a national or international consortium for pediatric oncology research could overcome all evaluation and validation issues so to improve the diagnostic use of miRNAs in RMS detection. Finally, all these studies were exclusively performed on RMS tissues or cells obtained by surgical or bioptic specimen, but a non-invasive approach, such as a blood sample, for these pediatric patients with RMS could be definitely improve their clinical management as RMS could be better monitored and detected. For this, liquid biopsy could be an optimal solution in where the use of plasma or serum could be able to unveil and confirm the diagnostic or prognostic role of miRNAs in RMS.

## 4. miRNA as Functional Players in RMS

**Figure 3:**
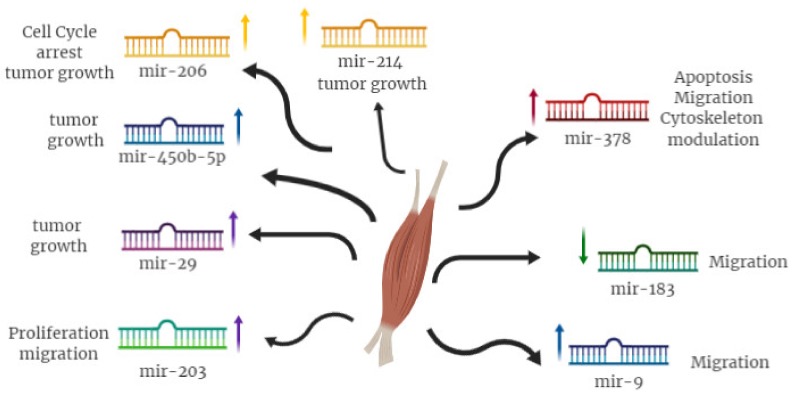
miRNAs modulation in RMS carcinogenesis.

As miRNAs play a crucial role in the development and progression of RMS, the possibility to exploit these molecules as new therapeutic targets in this pediatric tumor should be considered. To this matter, miR-378 over-expression was functionally demonstrated to cause changes in apoptosis, migration and viability through *IGF1R* down-modulation [3] (Figure 3). Moreover, replacement of miR-378a-3p induces cytoskeleton organization as well as modulation of muscle protein, such as *MyoD1, MyoR, desmin* and the myosin heavy chain [3]. MiR-183 knockout guinea pigs showed a reduction in tumor cell migration in vitro and stimulation of phosphatase expression in the tumor suppressor gene and tensin homolog (PTEN), which in turn favored the expression of early growth response 1 (*EGR1*), thus, strengthening the repression of cell migration [57]. Therefore, miR-183 plays an oncogenic role by targeting two tumor suppressor genes, *EGR1* and *PTEN* and miRNA deregulation is crucial to the development of several types of tumors. As for miR-9a, is able to inhibit cell migration and acts directly on *E-cadherin* [58].

To this regard, several approaches that up-regulate or down-regulate miRNAs have been used to characterize and identify the miRNAs involved in the RMS, demonstrating significant efficacy in the treatment of the pathogenesis of RMS following intravenous administration in vivo [60]. In particular, two pre-clinical studies have shown that ectopic expression of miR-206 by lentiviral vectors leads to cell cycle arrest and myogenic differentiation of RMS cells, preventing cell growth in vivo, thanks to inhibition of oncogene expression *c-MET* [53,54]. Furthermore, experiments with miR-183 knockdown mice led to significant reductions in tumor migration through the direct promotion of *EGR1* expression, regulator of cell migration [57]. Re-expression of miR-203 in RMS cells inhibits their proliferation and migration and promotes terminal myogenic differentiation by acting directly on p63 and leukemia inhibitory factor through JAK/STAT (Janus Kinases/Signal Transducer Activator of Transcription Protein) pathway modulation [61]. miR-29 re-expression in mouse model of RMS inhibited tumor growth through stimulation of differentiation mechanisms, suggesting a tumor-suppressor role for this miRNA [56]. Furthermore, in some studies, RMS growth and proliferation were significantly arrested by miR-450b-5p, strictly regulated by TGF-β1 [62]. Huang et al. demonstrated that overexpression of miR-214 was able to inhibit RMS tumor growth both in vitro and in vivo and induced myogenic differentiation by down-modulation of N-Ras [63].

A major weakness of miRNAs modulation studies in RMS is the employment of only commercially available RMS cell lines for in vitro approaches without a further in vitro validation with primary RMS cell lines generated from patients’ surgical specimen. An innovative way to bypass in vitro cell cultures could be the generation of in vivo models such as RMS Patients Derived Xenografts (RMS-PDXs). To date, few works reported the generation of RMS-PDXs, but none of them analyzed the potential of miRNAs in vivo in RMS development or for therapeutic purposes. Most of the functional studies did not consider the microenvironment role in RMS development that could influence cancer cells behavior and aggressiveness. Infiltrating immune cells have a critical and essential role in supporting tumor growth and this aspect should be also investigated to identify novel potential therapeutic targets for RMS management. The generation of innovative therapeutic agents combined miRNAs mimic or inhibitor with liposomes or nanoparticles, which could be a compelling challenge for RMS treatment for the next years. Although all reported data suggests a probable therapeutic role of miRNAs in RMS, unfortunately, the lack of pre-clinical data leaves unexplored such a critical issue that holds great clinical potential for this pediatric disease.

## 5. Conclusions

Genetic factors play an important role in the development of RMS. To date, molecular cytogenetics has characterized several genetic aberrations specifically involved in ERMS and ARMS; thus, a detailed comprehension of the molecular mechanisms underlying RMS tumorigenesis has not yet been achieved. In this review, we summarized the state of the art of miRNAs investigations in pediatric rhabdomyosarcoma. Several works emphasize how deregulation of miRNAs and their target genes is a crucial process for RMS. These small non-coding RNAs were described as involved in one or more cellular mechanisms and finely regulated in muscle cells. Small alterations in gene expression, even if they occur only at a miRNA level, could significantly influence the equilibrium between the pathological and physiological programs of cell fate and skeletal muscle development and differentiation. The potential use of miRNAs in RMS diagnosis, however, is still far from the clinics environment. The absence of data reinforcing miRNAs diagnostic value in large cohort of patients and lack of results on RMS blood specimens makes this issue still frail and in need of significant research. Another argument that requires intensive work is the cellular origin of miRNAs in RMS tumor tissues. Thus, knowledge of specific cell-type miRNAs is crucial to perform miRNA functional studies, still limited by the absence of preclinical models such as RMS PDXs. Despite all these limitations, the use of miRNAs as biomarkers in RMS diagnosis or, eventually, as therapeutic agents remains promising and unexplored.

## Figures and Tables

**Table 1:** Cytogenetic alterations in Rhabdomyosarcoma RMS.

Subtype	Cytogenetic Alteration	Chromosomes Involved	Genes Involved
Embryonal	Gains	whole chromosomes: 2,7,8,12,13, 19, and 20	*GOK*, *PTCH*, *TP53*
	Losses	whole chromosomes: 1,6,9,14,14	
	Loss of heterozygosity LOH	11p15.5, 11q, 16q	*IGF2*, *H19*, *CDKN1C*
	complex translocation	t(2;12;8)	*PAX3*
		t(2;20)(q35;p12)	*PAX3*
Alveolar	Rearrangements	t(2;13)(q35;q14);	*PAX3-FOXO1*
		t(1;13)(p36;q14), double minutes	*PAX7-FOXO1*
		t(2;2)(q35;p23)	*PAX3-NCOA1*
		t(2;8)(q35;q13)	*PAX3-NCOA2*
		t(X;2)(q13;q35)	*PAX3-FOXO4*
		t(8;13;9)(p11.2;q14;q32)	*FGFR1-FOXO1*
	Gains	12q13.3–q14.1 and 8p11.2–q11.2	*CDK4*, *MYCN*, *GLI*, *MDM2*, *FGFR1*, and *FGFR4*

**Table 2:** Myo-miRNAs in muscle development.

miRNA	Target Gene	Function
miR-1	*HDAC4*	Promote myoblasts differentiation
	*HAND2*	Inhibition of cardiomyocites proliferation
	*PAX3*	Inhibition of RMS proliferation
	*PAX7*	Promote muscle cells differentiation
	*CCND2*	Inhibition of RMS growth
	*cMET*	Inhibition of RMS development
miR-133	*SRF*	Promote myoblasts differentiation
miR-206	*PAX7*	Promote muscle cells differentiation
	*CCND2*	Inhibition of RMS proliferation
	*cMET*	Inhibition of RMS development
	*CX43*	Induction of myoblasts differentiation
	*Polα1*	
	*FSTL1*	
	*UTRN*	

**Table 3:** Non-myomiRNAs and their function in myogenesis.

miRNA	Target Gene	Function
miR-27b	*PAX3*	Inhibition of myoblast differentiation
miR-26a	*EZH2*	Promoter of myogenesis
miR-214	*EZH2*	Induction of myoblast differentiation
miR-181	*HOX11*	Induction of myoblast differentiation
miR-669a	*MyoD*	Inhibition of skeletal muscle differentiation
miR-29	*YY1*	Induction of myoblast differentiation
	*PAX3*	Inhibition of RMS proliferation
	*CCND2*	
miR-183	*PTEN*	Inhibition of RMS cell migration
	*EGR1*	
miR-203	*P63*	Inhibition of RMS proliferation
miR-9	*E-cadherin*	Inhibition of RMS migration
miR-450b	*TGF-β1*	Inhibition RMS development
